# Professionals' narratives of interactions with patients' families in intensive care

**DOI:** 10.1177/09697330211050995

**Published:** 2022-02-24

**Authors:** Anne M Nygaard, Hege S Haugdahl, Hilde Laholt, Berit S Brinchmann, Ranveig Lind

**Affiliations:** Department of Health and Care Sciences, 60482 UiT, The Arctic University of Norway, Tromso, Norway; Department of Public Health and Nursing, Levanger Hospital, 60500 Nord-Trøndelag Hospital Trust and NTNU Norwegian University of Science and Technology, Levanger, Norway; Department of Health and Care Sciences, UiT The Arctic University of Norway, Troms, Norway; Nord University and Nordland Hospital Trust, Norway; Department of Health and Care Sciences, UiT The Arctic University of Norway and Research Nurse at Intensive Care Unit, University Hospital of North Norway, Harstad, Norway

**Keywords:** Intensive care, critical care nursing, nurses, physicians, family-centered care, care ethics, narrative analysis

## Abstract

**Background:** ICU patients’ family members are in a new, uncertain, and vulnerable situation due to the patient’s critical illness and complete dependence on the ICU nurses and physicians. Family members’ feeling of being cared for is closely linked to clinicians’ attitudes and behavior.

**Aim:** To explore ICU nurses’ and physicians’ bedside interaction with critically ill ICU patients´ families and discuss this in light of the ethics of care.

**Research design:** A qualitative study using participant observation, focus groups, and thematic narrative analysis.

**Participants and research context:** Data were gathered from July 2017 to August 2019, in four ICUs in Norway through 270 h of fieldwork and seven focus groups with ICU nurses and physicians.

**Ethical considerations:** The Regional Committee for Medical and Health Research Ethics and the Norwegian Centre for Research Data approved the study. **Findings:** Quality of ICU family care depends on nurses’ and physicians’ attitudes, behavior, and personality traits. Three main themes were identified: *being attentive*, *an active approach,* and *degree of tolerance*.

**Discussion:** The findings are discussed in light of the ethics of care and empirical research from the intensive care environment.

**Conclusions:** This study shows that attentive, active, and tolerant clinicians represent a culture of ethical care that gives families greater freedom of action and active participation in patient care. Clinicians must not bear sole responsibility for this culture; it must have a firm basis in the hospital and ICU and be established through training, interprofessional reflection, and support of clinicians.

## Introduction

Critical illness has a significant impact on family members of ICU patients. They are in a vulnerable situation, often worried, insecure, and confused.^1–3^ Families are under undue pressure and are often anxious and at high risk of depressive conditions, including acute stress disorder, insomnia, complicated grief, and posttraumatic stress disorder.^4–6^

Clinicians’ attitudes and behavior will determine whether family members feel cared for, or alternatively, overlooked and offended. This relationship can be illuminated by the ethics of care, which states that humans in general are dependent on others.^7^ In an ICU family care setting the moral aspect lies in a (silent) demand that ICU clinicians act in the family’s best interest and are worthy of the family’s trust in them.^8,9^ This emphasizes the importance of clinician-family interaction in providing high-quality family care.

In this study, we decided to explore nurses’ and physicians’ approaches towards ICU patients’ families and elicit how family care is reflected in ICU clinicians’ everyday work. We aimed to examine ICU nurses’ and physicians’ bedside interactions with critically ill ICU patients´ families and discuss these in light of the ethics of care. This approach can enhance knowledge to improve the care of ICU family members.

## Background

Nurses play a pivotal role in ICU family care, being close to patients’ families throughout the ICU stay by providing them with information, comfort, and support and in organizing their presence and their contact with the ICU team.^10,11^ McAndrew et al.^10^ have identified facilitators and disruptors to nurse-promoted engagement with ICU families. Facilitators are at unit and organizational levels, a family adaptation level, and a nursing culture level. Disruptors are system barriers, ethical conflicts, family distress, and family exclusion.^10^ Although ICU nurses are responsible for the families’ daily ICU contact, physicians play a vital role vis-à-vis families regarding medical information and decision-making. The professional roles partly overlap and are mutually dependent.^12,13^ Family care quality therefore depends on good interprofessional cooperation and communication. A family-centered approach to healthcare is recommended in ICUs to mitigate families’ psychosocial stress and prepare them for decision-making and caregiving demands.^14^ Family-centered care involves a respectful and responsive attitude to families’ needs and values, and includes information sharing, participation, and collaboration.^14–16^ Family needs have previously been assessed using five dimensions: support, comfort, information, proximity, and assurance.^17,18^ However, focusing solely on families’ needs might make them passive care recipients, which may be considered an unintentional consequence of the family-centered approach. To date, more research has examined families as care recipients than as active partners.^19^ Although family-centered care is often highlighted, it seems to be challenging to implement in ICUs. Information on how it can be translated into daily ICU practice is lacking.^20^ Interaction between family members and clinicians must be explored to improve understanding of how clinicians influence family involvement.^20,21^

### Care ethics

Care ethics is an ethical approach that emerged in the 1980s, based on relational ontology.^22^ It is a context-bound approach, focusing on the interdependence and vulnerability involved in humans’ connection to one another. Different traditions of care ethics exist.^23–25^ In this article, we reflect upon our findings from a Scandinavian care ethics perspective. The Danish nursing professor Delmar emphasizes a care ethics “thinking horizon” in nursing in order to become more purposeful and attentive in practice.^7^ Delmar^7^ pursues the thoughts of the Danish philosopher Løgstrup and the Norwegian nursing philosopher Martinsen in addition to her own comprehensive empirical nursing research. According to Løgstrup, people are interdependent and cannot meet without being in a mutual relationship, where they “hand themselves over to each other.”^7,26^ An “ethical demand” is situated in this interdependence as an appeal emanating from the other to act in the other’s best interest.^27^ We have something of the other person’s life in our power. In a professional caring relationship, however, the power relationship is asymmetrical and care is unidirectional, based on the clinician’s solidarity and care for the weak.^28^ Care is related to promoting good, but equally importantly, preventing harm. A clinician–family relationship without care, such as when clinicians overlook or ignore relatives, will make relatives feel powerless and left out.

Traditionally, the ethics of care has primarily influenced nursing, not medicine. However, care and moral actions are not limited to nursing.^29^ Requirements for caring treatment are found in the Code of Ethics for Doctors,^30^ the ICN Code of Ethics for Nurses,^31^ and the Health Personnel Act.^32^ Although Delmar’s caring ethics^7,33^ is developed in nursing, this thinking horizon founded on relational ontology may also increase awareness and recognition of care as an ethical perspective in medicine.^34^

## Methods

This is a qualitative study with a narrative approach where the story is the object of the inquiry. Storytelling can provide understanding about human experiences. Narratives are situated in time, place, and a particular setting, and have “essential meaning making structures” where individuals or groups construct their identities.^35,36^ In this study, narratives were collected in a combination of participant observation and focus groups to elicit a clear understanding of clinician–family interactions. Participant observation provides an inner perspective to illuminate phenomena in their natural settings, while interviews provide comprehensive insight into participants’ experiences.^37,38^ Data were analyzed using Riessman’s^36^ thematic narrative approach.

### Setting and participants

Nurses and physicians from four Norwegian ICUs (6–18 beds) participated in the study. Three ICUs were in university hospitals with the highest level of intensive care, and one in a mid-range hospital. Recruitment took place orally or by e-mail via a contact nurse in each unit. All ICUs treated both surgical and medical patients, mostly adults. Most patients needed mechanical ventilation and were unable to express their wishes and needs. Visiting hours were flexible in two ICUs and fixed in the other two (3–4 h per day). However, exceptions were made when needed, for example, in end-of-life situations.

### Data collection

Data were collected from July 2017 to August 2019. First the researchers conducted two focus groups at the ICU in the mid-range hospital. In the other units, the researcher conducted fieldwork, followed by focus groups during the last week of observations. The fieldwork consisted of 11–14 shifts during 3 weeks in each ICU. Seven focus groups were conducted with 32 participants in all: three groups of nurses, two of physicians, and two of both nurses and physicians. NN_1_ performed the observations. A preliminary guide was developed with suggestions for observations: *how the family members were received, what was said, how the clinicians positioned themselves in the room, etc*. The fieldwork provided a unique opportunity to study the clinicians’ interaction, and enabled the researcher to ask questions at various points. Field notes with rich descriptions of clinician-family meetings were written during and after each shift. Following clinicians during their everyday work allowed for close contact with their reality. It enabled the examination of professional and interprofessional interactions and exchanges, both within and between professional groups.^20^

Each focus group was moderated by NN_1_ or NN_5_. A “question route”^37^ with open-ended questions was used and participants were encouraged to tell stories from their daily work with families. In addition, an observer (NN_1_, NN_2_, NN_4_, or NN_5_) paid special attention to the interaction between participants, took notes, asked supplementary questions, and gave an oral summary at the end. Conducting focus groups after the fieldwork enabled the moderator to ask in-depth questions about the observations. All interviews were digitally recorded and transcribed verbatim by NN_1_.

### Ethical considerations

The Regional Committee for Medical and Health Research Ethics (Ref. No.), the Norwegian Centre for Research Data (Ref. No.) and the participating ICUs approved the study. Before the fieldwork, the clinicians were informed by email about the purpose and scope of the study. NN_1_ also informed them orally on each shift. Written study information was posted at the ICU entrance and corridors to inform visiting clinicians and visiting family members. Further, since it was impossible to observe clinicians' work without simultaneously observing patients and relatives, the researcher also informed conscious patients and visiting family members and requested their permission to observe in the patient’s room. None refused this request. Written informed consent was obtained from the focus group participants. Field notes and transcriptions were anonymized.

### Data analysis

In thematic narrative analysis, researchers focus on keeping the stories “intact” and analyzing them separately to elicit themes. Themes refer to the meaningful “essence” in the dataset.^39^ According to Riessman,^36^ in thematic narrative analysis, the content of each story is the exclusive focus. Instead of theorizing across cases, the researcher keeps the story intact by theorizing from each case.^36^ NN_1_ worked on the narratives one by one, studying and reading them several times, searching for preliminary themes that illuminated clinician–family interactions. NN_1_ and NN_5_ discussed and refined the preliminary themes and presented and discussed these with the entire research team. All stories were then compared to identify common themes. Specific narratives have been selected to illustrate the themes.

## Findings

The analysis shows that ICU family care varies in quality, depending on the individual clinician’s attitudes, behavior, and personality. Participants themselves stated that their family care depended on their personality and use of discretion. Three themes were identified: (1) *being attentive*, (2) *an active approach,* and (3) *degree of tolerance*. The narratives presented under each theme reveal contrasts in clinicians’ interaction with families.

### Being attentive

Being attentive describes ICU clinicians’ ability to pay attention, be considerate, listen, concentrate, and be alert and their perception of families’ verbal and non-verbal communication.

Lisa, an ICU nurse, is responsible for a patient in her 70s, admitted for several weeks with severe respiratory failure. She has a tracheostomy. Sometimes she can breathe without a ventilator, but weaning is very difficult. She is alert and can speak with a tube when breathing without a ventilator. She tells Lisa that she doubts if she will recover, she is tired, and the dyspnea attacks make her frightened and desperate. Lisa knows her medical history well and realizes how serious her respiratory failure is.

One day when the patient’s family visit, Lisa suspects that they do not really understand how sick she is. Several times they tell her: “You’ll soon get better and come home”. Later, Lisa asks her: “Does your family understand how sick you are?” “I don’t think so,” she replies. “That’s what I suspected,” says Lisa. The patient says that it is difficult to talk to her family about how she is, her thoughts about not living much longer, not wanting to exercise or “fight” to get off the ventilator. Lisa looks at her, listening closely. Later, Lisa discusses her concerns with Eric, the ICU physician. When he sees the patient, he talks to her for some time and finally she repeats to him what she said to Lisa. She feels like giving up. But she also says she has a lot to live for, a husband, children and grandchildren. She is worried about her husband, how he will manage alone. “We can help you to talk to your husband,” says Eric. He speaks in a calm voice, looking straight at her, and takes his time. Lisa is sitting with them, listening to them, making comments and nodding.

(Field note, ICU 3)

The nurse’s sensitivity to the patient’s weak body and words enables her to remain close to the patient’s suffering and carefully ask her if she wants to talk about how her family views the situation. Due to her concern about the patient’s burden, she discusses it with the physician, and together they support the patient in planning how to communicate this sensitive information to the family. A few days later, during a talk with the family, their suspicion of the family’s lack of understanding is confirmed.

The patient and her husband are sitting close together, holding hands. Eric first talks about the disease. Quite soon the patient exclaims: “I’ve lost all hope”. Her husband is horrified, looks at her and says: “No, you mustn’t give up!“. The patient says she is afraid and thinks she cannot get out of the situation. Her husband sits quietly with tears in his eyes.

(Field note, ICU 3)

An attentive clinician, like Lisa and Eric, realizes what is most important in this situation. The fieldwork shows that family members’ concerns are often expressed as hints. Instead of asking directly, they may ask vague, indirect questions. “So he’s wearing a different oxygen mask today?” may express a worry that the patient’s condition has worsened. “I can’t sleep at night” may mean that the family member is upset. Body language such as “an unfocused look” or sitting “on the edge of the chair” can reveal distress or anxiety.

The opposite of attentive clinicians is those who are bad listeners, make sudden movements, speak sharply, and do not get the details of what is said. They can do patient-oriented work quickly and efficiently, but pay little attention to family members, do not get their hints and thus do not respond to them. In ICU 2 the following situation occurred:

The patient’s brother and sister-in-law are visiting. Hans (the nurse) says hello, then turns his back on them and sits down at the computer. The visitors stand by the bed; there are no chairs. They talk to the patient, but he is tired, does not feel like saying much, closes his eyes and the conversation ends. They stand still, look around and comment on all the “equipment” around the bed. Then it is quiet again. After a few minutes, the sister-in-law says: “I’m glad it was such a success”. Hans does not look up from the computer or respond to them. A few minutes later, the visitors say goodbye to the patient. Then Hans turns round and says goodbye before they leave.

(Field note, ICU 2)

As Hans has his back to the visitors, he does not realize they want contact. They are left to themselves and receive no confirmation that the patient is doing well. Without any response, they “give up” and leave. Maybe Hans means well, perhaps he wants to give them time alone with the patient, or he may think they are talking to each other. But he is not attentive; he makes no attempt to find out what they really need or whether he has interpreted the situation correctly.

### An active approach

Just as clinicians are attentive to different degrees, we also see differences in how active they are towards family members. An active approach involves asking questions, making arrangements for families, and including them in patient care. It is demonstrated by both words and actions.

A young man is admitted to the ICU. Oscar, the nurse, is told that he will have a CT scan in 30 min. Oscar rings the patient’s father to mention the scan. “I just wanted to tell you in case you were coming here,” he says. They agree that the patient’s father will come in 1 h, when the scan is over.

The father arrives at the agreed time, gives Oscar a hug, walks over to the bed, looks at his son, touches his arm and strokes his cheek. Oscar is on the other side of the bed. The patient’s father says he thought a lot about the information he got the previous evening and slept badly that night. Oscar looks at him, listens, says a few words, but lets him speak. The father has tears in his eyes, his voice is trembling. Oscar explains calmly, precisely and in simple language about the patient’s situation now and last night. He also talks about feelings that family members can get in such serious situations, and the visitor talks about how he feels. While they are talking, the physician in charge of treatment arrives. Oscar finishes the conversation and sits down with the physician. They talk quietly while the father is still standing by the bed. He sometimes looks at the doctor and Oscar. Oscar notices this, gets up and goes back to the father. The physician sits at the computer reading the patient record. Oscar tells the father: “If you have any questions, you can ask him [the physician]”. The physician gets up, walks over to them, and greets the father. (Field note, ICU 4)

Oscar’s active behavior and actions show his care and understanding of the father’s situation. He stands by the bed with him, they have eye contact, and he is friendly and accessible. He is proactive in informing the father about the CT scan, providing other important information, and inviting him to come straight in to the ICU. He listens to the father’s concerns before speaking, but also makes suggestions and talks about the patient’s condition and treatment and about typical thoughts and feelings of family members. His language is direct and simple. Telling the father that he can talk to the physician includes him in the conversation and ensures that he receives the latest information.

The opposite of active clinicians is passive clinicians. They do not ask questions, make suggestions, or act actively; they wait for the family’s questions, avoid eye contact, and pay little attention to the family.

An old patient who is sedated and mechanically ventilated is visited by his wife and daughter. They sit at the end of the bed while the physiotherapist moves the patient’s arms and legs. The nurse, Sophie, with her back to the visitors, is busy checking the infusion pumps. The patient’s wife puts her face in her hands and sobs quietly, and her daughter hugs her gently. They talk quietly. Sophie sits at the computer without looking at the visitors, updating the patient record.

The visitors follow the physiotherapist’s work. When she lifts the patient’s injured leg, the wife leans over to look. Neither the physiotherapist nor Sophie say anything about this, perhaps they do not notice it, as they talk about the patient’s injuries. The visitors look at the physiotherapist and Sophie. The physiotherapist finishes and talks to Sophie about further treatment without looking at the family members or including them in the dialogue. The physiotherapist leaves and Sophie continues at the computer. The visitors walk over to the patient and look at him. The daughter asks Sophie if her mother can touch the patient. “Yes, but don’t stroke his arm, that might be unpleasant,” she replies quickly and sharply. (Field note, ICU 4)

Sophie is not attentive to the visitors. She avoids eye contact and “does her job.” Her passive approach is seen in her lack of response to the family members. They are not involved or informed, and are actually reproached when they suggest touching the patient. The nurse’s passivity is also reflected in her position in the room. Unlike Oscar, who stood by the bed with the patient’s father, Sophie sat by the computer or stood with her back to the family members. The findings from the fieldwork are supported by stories nurses and physicians told in the focus groups: some clinicians have a more active approach than others.

One nurse says that she rings relatives if they have not visited the patient for several days. She asks how they are and why they have not come. Can she help them with anything? The other nurses in the focus group are surprised - they do not usually do that.

(Focus group 7, ICU 4)

The active–passive dichotomy is also evident in clinicians’ communication with families and how they start the conversation. Active clinicians ask how family members are, if they have any questions, and if they are worried about anything. They also ask them to describe the patient before the illness. An active approach involves letting the family sit by the patient’s bed, showing them how to hold the patient’s hand, telling them that they can talk to the patient even if he/she cannot reply, and explaining about the equipment and ICU procedures. Passive clinicians mostly talk about the patient’s current condition, temperature and blood sample results, and only provide other information when families ask for it.

### Degree of tolerance

Clinician–family interaction is also expressed through nurses’ and physicians’ degree of tolerance towards families. This seems to depend on clinicians’ workload, stress threshold, robustness, knowledge, and experience.

Data from fieldwork and focus groups showed that several young, newly trained ICU nurses had difficulty in concentrating on necessary patient tasks when families were present. ICU treatment is advanced, making it difficult to take care of family members simultaneously. Trainee physicians said that as new staff they found it challenging to be “thrown into” difficult conversations with families. Younger, less experienced clinicians could also make relatives feel insecure and afraid.

A patient’s wife talks about what happened the previous night. When she and her son arrived, her husband was very stressed and agitated. They were worried at seeing him in that state. The wife thought the physicians who came to see the patient were “so young, and they said nothing. We got so stressed. Luckily, an experienced doctor came and gave us good information”.

(Field note, ICU 3)

Older ICU clinicians said that over the years they had gained more experience and knowledge to enable them to relax more with families. When newly graduated, they were less sure about what to say, and how to help and comfort families.

Susan has been an ICU nurse for over 20 years. She is responsible for an old patient, acutely admitted a few hours ago. His condition is severe and unstable. Soon after Susan started her shift, the patient’s wife, daughter and grandson arrived. Susan greets them at the door. She puts out chairs and gives the patient’s wife the chair closest to him, telling her that she can hold his hand. She offers them something to drink. Then she talks about the patient’s condition, the treatment and the equipment. Later three more relatives arrive. Despite the ward’s fixed visiting hours and limited numbers of visitors, Susan welcomes them and fetches more chairs. They all sit around the bed, sometimes talking, sometimes quiet. The atmosphere is peaceful. Susan sits in the background and tells her colleague: “I think this is a nice situation”.

(Field note, ICU 3)

Susan is confident and very capable. She can combine caring for a critically ill patient and many family members. She is flexible and ignores the visiting regulations by exercising discretion to meet the family’s needs.

Sound knowledge of critical illness and family care gives clinicians confidence when informing families about ICU patients’ complex conditions. Oscar, a nurse, said:

“… it’s very important for me to have good knowledge, understand the patient’s disease, know the pathophysiology and understand the intensive care provided so I can explain this clearly and simply to families”.

(Field note, ICU 4)

However, more experience and knowledge does not necessarily mean better family care. Several participants said that older, experienced nurses could be less flexible and tolerant than younger ones. One nurse said: “*I was better before, now I get tired of them* [families]”, while another stated: “*I’m so old, I can’t face having relatives around so much*”. Younger nurses often showed more commitment and flexibility. Regardless of age and experience, the degree of tolerance was clearly seen in clinicians’ behavior towards families. It varied between the extremes of calm, polite, supportive, and inclusive clinicians and abrupt, rude, critical, and dismissive ones.

Participants also stated that caring for critically ill patients over time affected their view of what was really serious. They had experienced so many situations and observed and treated so many ICU patients that they had developed a higher tolerance threshold for what was serious or critical. One ICU physician described this as “*speed blindness.*” A patient situation that seems straightforward to clinicians may be perceived as very serious by families. Several participants also found that the ICU workload was increasing. In one focus group, a nurse described how nurses must go straight “*from end-of-life care to receiving a new patient.*” Clinicians missed time for reflection and talking to colleagues, and several became exhausted over time. However, some family members affected clinicians’ emotions in a positive way, motivating them to “*go the extra mile.*. The clinician–family relationship or alliance will affect how much families can be present and involved. Eric, a physician, and Marie, a nurse, discussed this in a focus group.

Eric: If you feel that families trust you, you’re relaxed and then it’s ok having them present during a procedure. Then there are those where the alliance has been bad from the start and then I wouldn’t take the risk; if they’re present, I’d have to focus on them so I don’t make mistakes … Marie:… I think it’s like that for nurses too. Having a good relationship with the family is fine. But if they’re always asking questions, the “wrong questions”, it’s a bit harder to work … I feel there’s a difference at least. With some relatives it’s fine, they can be present any time, but with others you just think: “Oh… hope time passes quickly, I want to finish”. Eric: …then there are some anxious ones, neurotically wanting to know everything… they need lots of attention and you can’t keep focused… Marie: …sometimes there are relatives it’s really hard to get along with, you have nothing in common…, no chemistry, while others… you could “invite them home”… it’s kind of strange…

(Focus group 5, ICU 3)

Split families with conflicts are viewed as challenging, as are those who are insistent, critical, and ask many questions. There can also be big differences between individual family members. One may be very demanding, while another is very satisfied or very frightened. Clinicians’ views of families also vary; one may find a person “tiresome,” while another feels very sorry for the same person.

## Discussion

The main findings are that ICU clinician–family interaction is related to whether clinicians are *attentive, active,* and *tolerant* towards relatives. This affects care provision and families’ freedom of action, which again influences the quality of family care. ICU families expect clinicians to help them handle the new and confusing situation they have been “thrown into.” According to Løgstrup, certain phenomena are fundamental to human existence, such as trust, openness, compassion, mercy, and hope.^33^ Showing trust is not a choice people make, but an existential phenomenon we are thrown into.^27^ However, one may be rejected when reaching out to another person. Thus, in an ethical sense, people are not primarily independent of others and the community.^9^ Empirical research, however, reveals a tendency for individualist, liberal values like “autonomy,” “independence,” and “self-management” to dominate patient and family care, especially in Western countries.^26^ Being independent of others’ help is also closely connected to integrity and dignity.^7^ Even if these liberal values are vital to our modern life, they may have negative outcomes for patients and their families.^7^ The fieldwork shows how some clinicians left families to themselves at the patient’s bed, failed to inform them, or ignored them. Wong et al.^3^ state that families described such experiences as being kept in the dark. Such lack of care can cause harm and represents an important moral concern from a care ethics perspective.^29^

Attention is a core quality in ICU practice. ICU patients’ severe condition requires continuous monitoring and assessment.^13^ Being attentive is a filtering process where nurses *“separate things of particular significance from less significant things.”*^27,40^ It requires an intense presence, concentration, and perception of the situation.^27^ To ensure safe and high-quality care, bedside nurses must be sensitive to every slight change in the patient’s condition and the uniqueness of each situation.^41^ Traditionally, ICU clinicians have focused on patients much more than their families.^42^ Today, a holistic patient- and family-centered care approach is emphasized and the importance of caring for and involving the family is acknowledged.^19,43^ ICU clinicians need to look beyond all the equipment and physical parameters towards the unique needs of the patient and family.^43^ Family members’ feelings are in turmoil in the ICU setting,^2^ but unfortunately many report poor emotional support from ICU clinicians.^44–47^ The ICU environment is complex, event-driven, and time-pressured.^48^ Several reasons have been suggested for clinicians’ lack of support, such as their intense focus on medical care, poor communication and interpersonal skills, and insufficient training in meeting family members’ emotional and mental needs.^45,46,49^ This was confirmed by the focus group participants, who also mentioned the increased workload and minimal time for discussion and reflection. Bedside nurses may feel torn between treating the patient as their highest priority and caring for the patient’s family.^50^ However, this study shows how inattention can lead to families’ needs being neglected or overlooked. Further, our data demonstrate how clinicians must be attentive to family members’ body language, vague hints, and covert questions. ICU clinicians’ ability to respond to relatives’ concerns is considered supportive and comforting.^49^ In the ICU, many family members are afraid of being a nuisance, and are unsure of their caregiver role.^45,49^ Being attentive requires sensitivity as a response to a silent demand. An attentive clinician may open up, listen to the unspoken words, and with imagination based on humanity and insight act responsibly and actively as needed.

Clinicians’ attitudes towards family involvement in patient care may be barriers or facilitators.^11^ This is evident in the narratives describing nurses’ and physicians’ active and passive approaches to families. While some nurses spontaneously gave families information and encouraged their involvement, others waited for them to express their needs. This shows clinicians’ position of power and gatekeeper role, allowing them to decide whether to include relatives. Families are completely dependent on clinicians’ decisions and use of power. The power relationship between the two parties is asymmetrical.^8,26^ Nurses’ and physicians’ authority is obvious in their position, uniform, professional knowledge, and personal behavior.^26^ Clinicians are probably also subject to a silent demand to diminish the power imbalance and become attuned to the world of the family members.^27^

Gatekeeping depends on discretion, but also on the individual clinician’s mood and personal energy.^51^ This concurs with our findings showing that clinicians’ tolerance and care of family members is affected by their knowledge, professional experience, personal attitudes, behavior, and stress tolerance. Clinicians’ professional and personal qualities seem interwoven and difficult to distinguish. According to Page et al.,^50^ nurses consistently oscillate between their personal and professional selves. Clinicians can be too close and overprotective but also too distant and paternalistic.^7^ An excessively close relationship can become too dependent, leaving families little freedom. A too distant relationship may leave families to themselves without the care they need. Both extremes may imply that clinicians overlook what really matters to a family in a particular situation.^7,8^ Professional care differs from private and personal care in requiring professional knowledge and empathetic skills, but also the ability to distinguish between one’s own and the family’s needs.^27^ Awareness of one’s own feelings through reflection can help clinicians avoid too close, sentimental feelings or too much distance, which may make care more personal than professional.^8^

This study shows that clinicians’ engagement and support for families depends on their degree of tolerance. Some family members are considered particularly difficult, such as those who are very critical, anxious, or emotional. This affects the chemistry and thus communication between clinician and family. Clinicians adapt their communication with families to their ideas of acceptable behavior and the norms and priorities of the ICU.^52^ Several studies have identified poor communication between ICU clinicians, especially physicians, and family members.^53–55^ Leslie et al.^52^ argue for developing communication strategies suitable for all types of relatives. Fortunately, communication is now more in focus, and several studies show promising results of training in communication strategies/skills for nurses and physicians.^53,54,56^ Nevertheless, there is a tendency to reduce many of the problems of interaction between physician and patients/family members to poor communication skills even when the (real) problem is to connect with patients/family and understand their needs.^29^ This reflects the importance of a care ethics approach focusing on people’s interdependence as well as moral attentiveness and contextual sensitivity in relation to how clinicians gain knowledge to act morally.^29^

The findings suggest that ICUs lack a common interprofessional ethical culture for family care. Responsibility for families seems to fall mainly on individual clinicians, particularly nurses, which can cause great variation in family care quality. However, the individual clinician should not have sole responsibility.^10,11,14^ Nurses should take a leading role in family care^10,19^ and many factors supporting nurses’ ability to promote family engagement in the ICU have been identified.^10^ Hospital management must facilitate consistent family care. This includes establishing an ICU nursing culture that promotes nurses’ moral resilience and enables them to enhance their family nursing skills. If family care is valued and emphasized in the ICU nursing culture, nurses are more likely to promote and prioritize such work.^10^ Family care is also dependent on good nurse–physician collaboration.^19^ To enhance care ethics thinking, clinicians should reflect on specific clinical situations to improve their attentiveness and judgment.^7,26,34^ Such interpersonal skills should also be key topics in ICU specialist education in addition to technical and medical knowledge.^46^ Importantly, ICU management needs to initiate closer interprofessional collaboration on specific guidelines for family care.^57^

This study shows that attentive, active, and tolerant clinicians represent a culture of care ethics that enhances families’ freedom of action and active participation in patient care. Yet this ethical challenge is little discussed and acknowledged, especially in medical ethics.^29^ In nursing, care ethics is more acknowledged^29^ but our study shows that even here this perspective needs greater implementation. It is necessary to challenge and supplement the prevailing ideals of detachment and non-interference in medicine.^29^ A care ethics approach also aligns with the principles of patient- and family-centered care that emphasize mutually beneficial partnerships among clinicians, patients, and families.^15^

### Limitations

Participant observation combined with focus groups resulted in rich data in the form of narratives. Particularly during fieldwork, the researcher was close to ICU clinicians’ interactions with families in specific clinical situations. A further strength was the inclusion of both physicians and nurses; although ICU family care is traditionally associated with nursing, physicians also play a vital role, as seen in this study. An additional strength would be to include family members as participants, to verify the researchers’ findings that clinicians’ attentiveness, active approach, and tolerance are essential for families to feel cared for. However, the research group did discuss the findings with patient representatives, a former ICU relative, and a former ICU patient. The former ICU relative is also a qualified researcher, has participated in the entire research process and is a co-author (NN_3_).

## Conclusion

High-quality ICU family care that includes family members as active partners in patient care depends on clinicians focusing on families’ wishes and needs and being active and tolerant towards families. This represents ethical family care. However, ICU clinicians’ family care seems to be largely based on the individual clinician’s personality and experience, rather than evidence-based guidelines and a common culture in the unit/hospital. As the study shows, family care is an individual professional responsibility, but it is also a key responsibility of the healthcare organization through training, interprofessional reflection, and support to clinicians to create a common culture of ethical family care in the ICU.
